# Transcriptomic, Metabolomic, and Physiological Analyses Reveal That the Culture Temperatures Modulate the Cryotolerance and Embryogenicity of Developing Somatic Embryos in *Picea glauca*

**DOI:** 10.3389/fpls.2021.694229

**Published:** 2021-09-01

**Authors:** Ying Cui, Ying Gao, Ruirui Zhao, Jian Zhao, Yixuan Li, Shuaizheng Qi, Jinfeng Zhang, Lisheng Kong

**Affiliations:** ^1^Beijing Advanced Innovation Center for Tree Breeding by Molecular Design, College of Biological Science and Biotechnology, Beijing Forestry University, Beijing, China; ^2^Department of Biology, Centre for Forest Biology, University of Victoria, Victoria, BC, Canada

**Keywords:** cold acclimation, culture temperatures, cryo-tolerance, embryogenicity, gene expression, metabolites, *Picea glauca*, somatic embryo

## Abstract

Cryopreservation is one of the key technologies for the mass propagation of conifers via somatic embryogenesis. Cryotolerance and embryogenecity of conifer somatic embryos (SEs) could be affected by different temperature treatments, for which the underlying mechanisms were unknown. In this study, the developing SEs of *Picea glauca* obtained their cryotolerance with a survival rate of 100% when cultured on maturation medium at either 23°C for 4 weeks or 4°C for 10 weeks. However, only the embryos that underwent 4°C acclimation remained high embryogenicity, i.e., 91.7% based on cryovials or 29.3% on the plant tissue. Analysis of differentially expressed genes (DEGs) revealed that both 23 and 4°C treatments led to drastic changes in the gene expression, i.e., 21,621 and 14,906 genes, respectively, and the general increase in many oligosaccharides and flavonoids, in addition to the content change of proline (1.9- and 2.3-fold at 23 or 4°C) and gallic acid (6,963- and 22,053-fold). There were 249 significantly different metabolites between the samples of 23 and 4°C treatments and the changing trend of the sorbitol, fatty acids, and monosaccharides differed between these samples. During 4°C-acclimation, the metabolites of the arginine biosynthesis pathway increased between 2.4- and 8.1-fold, and the expression of antioxidant genes was up-regulated significantly. At 4°C, the up-regulated genes were for germ-like proteins, instead of seed storage proteins at 23°C. Concentrations of abscisic acid and jasmonic acid increased up to 2- and 1.5-fold, respectively, in the cold-acclimated embryos. After 10 weeks at 4°C, the embryos stayed at pre-cotyledonary stage with 17.1% less DNA methylation and fewer storage substances than those at 23°C for 4 weeks, which developed cotyledons. This research provides new insights into mechanisms underlying the response of SEs to different culture temperatures and benefits method development for germplasm conservation in conifers.

## Introduction

Somatic embryogenesis is the process by which somatic cells are reprogramed to embryogenic cells, and later undergo many morphological and biochemical changes to produce somatic embryos (SEs) (Quiroz-Figueroa et al., 2006). Due to the bipolar structures of SEs with both shoot and root apical meristems, they are ideal materials for plant micropropagation (Isah, 2016). Somatic embryogenesis (SEis) has demonstrated many advantages compared with some other clonal propagation methods including rooting of cuttings and organogenesis, such as high propagation efficiency, ease of rooting, automatic production, and long-term cryopreservation (Bonga, 2015). This led to the wide usage of SEis in clonal forestry. Additionally, it is an ideal regeneration system for genetic transformation and germplasm preservation. Technology development of SEis in coniferous species started in 1985 (Chalupa, 1985; Hakman et al., 1985), which led to the use of SEs on the large-scale production of somatic seedlings in a few conifers (Stasolla et al., 2002; Egertsdotter et al., 2019).

Cryopreservation is the key technology during SEis. Cryopreservation preserves embryogenic cells or tissue at ultra-low temperature, such as in liquid nitrogen (LN), which was about −196°C (Park, 2014). During cryopreservation, the embryogenic cell or tissue can maintain its genetic stability and the juvenility of the donor tissue for a long time. Therefore, cryopreservation is an ideal method for the long-term storage of elite embryogenic cells or tissues (Cyr, 1999). In a typical mass-production system, part of the embryogenic tissue is cryopreserved in LN before the process of genotype test that is used to select elite genotypes based on tree performance in the field. After the test, which usually takes a few years, tissues of the selected elite genotypes are taken out from cryo-tanks, thawed, and used for production (Klimaszewska et al., 2016). Cryopreservation is less in labor intensity and contamination risk compared with the maintenance of embryogenic tissue by regular sub-cultures. Furthermore, the embryogenic capability of plant tissue might decline or even be lost during a long-term sub-culture, while cryopreservation could maintain the embryogenic capability of the embryogenic cell or tissue (Malabadi and Nataraja, 2006). Therefore, cryopreservation of plant tissue in LN is a commonly employed technology for the long-term storage of embryonic cell lines (Matsumoto, 2017).

Conventional methods for cryo-preserving conifer embryogenic tissue need treatments using osmotica and protective agents, usually dimethyl sulfoxide (DMSO) and a slow cooling process (Salaj et al., 2012; Latutrie and Aronen, 2013; Carneros et al., 2017), to overcome cell membrane damage caused by ice crystal formation and cell dehydration under the ultra-low temperature. In this case, remarkable genetic variation could be detected in DMSO-treated embryogenic tissue (Aronen et al., 1999) or seedlings originated from DMSO-treated tissue through conventional methods of cryopreservation (De Verno et al., 1999). It is better to avoid using any toxic cryoprotectant in the procedures of cryopreservation (Tessereau et al., 1994). In order to optimize cryopreservation methods, attempts have been made to control DMSO usage to a minimum dosage and/or with the shortest time period of its treatment. In the study of Kong and von Aderkas (2011), they reported a novel cryopreservation method that was applied successfully to embryogenic materials of interior spruce and Douglas-fir. This method is simple without using any amount of DMSO. In this novel method, embryogenic tissue was inoculated on embryo maturation medium and cultured in the darkness at 23°C for a couple of weeks and then transferred to 5°C for several weeks. Following this treatment, immature SEs of the tissue obtained cryotolerance and remained high embryogenicity, which is the ability to initiate embryogenic tissue. The process in which non-freezing low temperatures leading to higher freezing tolerance in plants is usually referred to as cold acclimation. Thus, cold acclimation of immature SEs at 5°C for several weeks is one of the key factors in the novel cryopreservation method developed by Kong and von Aderkas (2011). Cold acclimation involves distinct changes in gene expression, protein synthesis, and metabolite profiles (Renaut et al., 2006; Ding et al., 2019; Leuendorf et al., 2020). However, there is little information available about the responses of developing SEs to culture temperatures. Except that, some Norway spruce SEs reaching a certain developmental stage were reported of survival at a freezing temperature by secreting antifreeze-like proteins (Sabala et al., 1996). In addition, the desiccated mature SEs with a relative water content of approximately 0.13 were reported surviving in LN without additional cryoprotectants in white spruce and interior spruce (Percy et al., 2001). The mechanisms underlying the cold (4°C)-induced cryotolerance and the maintained embryogenicity of the SEs remained unknown.

In this study, the new cryopreservation method according to Kong and von Aderkas (2011) was tested during white spruce (*Picea glauca* [Moench] Voss) SEis. Then the transcriptomic, metabolomic, and physiological analyses were performed to study the responses of a somatic embryo (SE) to different temperatures and to explore the underlying mechanisms, especially for the induced cryotolerance and the affected embryogenicity of the embryos. Our transcriptomic, metabolomic and physiological analysis revealed the common and the different mechanisms in obtaining cryotolerance between 23 and 4°C treatments and also the factors retaining embryogenicity of the treated plant materials. The findings of this study will further our knowledge on the responses of plant embryos to environment temperatures at the molecular and physiological levels. Our results could benefit technology optimization for germplasm preservation.

## Materials and Methods

### Plant Materials

Embryogenic tissues were induced from embryos dissected from mature seeds of white spruce according to the study of Tremblay (1990). The induction medium was a half-strength modified Litvay's (1/2 mLV) medium (Litvay et al., 1985; Kong and von Aderkas, 2007) supplemented with 2 mg/L 2,4-dichlorophenoxyacetic acids (2,4-D), 1 mg/L 6-benzyl amino adenine (6-BA), 0.8 g/L hydrolyzed casein, 0.5 g/L glutamine, 10 g/L sucrose, 3 g/L gellan gum (P8169, Sigma-Aldrich, St. Louis, MO 63103, USA). The mature seeds were sterilized for 1.5 min in 75% alcohol, and then for 10 min in a 50% (v/v) solution of commercial bleach (sodium hypochlorite, 5.5–6.5% available chlorine). After sterilization, seeds were rinsed three times with sterile distilled water. Embryogenic tissues were induced from the mature embryos about 40 days after culture initiation. The embryogenic tissue from each embryo was named as a cell line (genotype), transferred to the maintenance medium (1/2 mLV medium supplemented with 1 mg/L 2,4-D, 0.5 mg/L 6-BA, 0.5 g/L hydrolyzed casein, 0.5 g/L glutamine, 10 g/L sucrose, and 3 g/L gellan gum), and sub-culture once two weeks. After two sub-cultures, 2 g of embryonic tissue was inoculated into 100 ml of the liquid maintenance medium (maintenance medium without a gelling agent), cultured in a shaker at 100 rpm, 23 ± 1°C. Embryogenic tissue was transferred to the liquid pretreatment medium when fully dispersed (1/2 mLV medium added with 30 μM abscisic acid (ABA), 0.4 g/L hydrolyzed casein, 0.5 g/L glutamine, and 10 g/L sucrose) and cultured under the same conditions for 1 week. After pretreatment, embryogenic tissues were placed onto the maturation medium (1/2 mLV medium supplemented with 45 μM ABA, 0.2 g/L hydrolyzed casein, 0.4 g/ L glutamine, 30 g/L sucrose, 10 g/L maltose, and 6 g/L gellan gum) and cultured at 23 ± 1°C in the dark to stimulate embryo maturation. After comparing the embryogenic capability of different embryogenic tissues, the white spruce cell line 3 (WSP3) with high embryogenic capability was selected and used for all the experiments in this research. All mediums in this research were adjusted to pH 5.8 before autoclaving.

After embryogenic tissue of WSP3 was placed on the maturation medium and cultured at 23 ± 1°C, materials were sampled at 0, 2, and 4 weeks, and designated as MMA0, MMA2, and MMA4, respectively. For cold acclimation, the immature SEs were placed in the incubator with a temperature of 4 ± 1°C after a 2-week culturing on the maturation medium at 23 ±1°C. Plant tissue during cold acclimation was sampled at 2, 4, and 10 weeks and named MCA2, MCA4, and MCA10, respectively. Samples collected at each time point included three independent replicates for subsequent analysis.

### Cryotolerance Tests

Plant materials after the treatments were placed into cryo-vials, respectively. Each vial contained 600 mg plant tissue approximately. The vials were immersed directly into LN and stored in the cryo-tank for 24 h before being thawed in a water bath at 38°C for 5 min. The plant materials were then cultured on an induction culture medium at 23°C in dark. Plant tissue of 200 mg fresh weight (FW) was used for each treatment, with six replicates. The tissue (ca. 200 mg) was placed on an induction medium and dispersed into 15–20 pieces in a Petri plate. Tissue survival rate and embryogenicity were evaluated 30 d later according to the method described by Kong and von Aderkas (2011). The embryogenic capacity of the newly initiated embryogenic tissue from the treated materials was evaluated by putting about 0.1 g embryogenic tissue on the maturation medium and counting the number of SEs after 30 d of maturation. During evaluation of the embryogenic capacity, six repeats were performed.

The triphenyl tetrazolium chloride (2, 3, 5-TTC) assay was carried out according to the study of Towill and Mazur (1975). Plant tissue of 50 mg was collected from each treatment with six repeats. As a control, three repeats of each treatment were directly immersed in 500 μL 0.5 M TTC (dissolved in pH 7.5 sodium phosphate buffer) and incubated in dark for 24 h at 23°C. The other three repeats went through the cryo-and-thawed process before the TTC assay. The red color reaction of the tissue was examined under a dissecting microscope.

### Transcriptome and Metabolome Analysis

Ribonucleic acid sequencing and detection of widely targeted metabolites were performed by Metware Biotechnology Co., Ltd (Wuhan, China). Total RNA was isolated from samples of each treatment with three replicates for RNA sequencing. Quality and concentrations of RNA were determined by electrophoresis, Nanophotometer (Micro Drop, Shanghai Bio-DL Scientific Instrument Co., Ltd., China), and Agilent 2100 bioanalyzer (G2939A, Agilent, USA). Ribonucleic acid sequencing (RNA-seq) libraries were constructed according to the laboratory protocol (Metware Biotechnology Co., Ltd.) and RNA was sequenced using the Illumina Hiseq × TEN (USA). After the adapters and low-quality sequence reads were removed from the raw reads, the clean reads were assembled with Trinity v2.6.6 (Grabhher et al., 2011), which were used as the reference sequences. The longest cluster sequences were obtained as unigenes after Corest clustering of the assembled transcripts. Thereafter, the annotation of the unigenes was carried out by blasting them in KEGG (Kyoto Encyclopedia of Genes and Genomes, http://www.genome.jp/kegg/), NR (NCBI Non-Redundant Protein Sequences, https://ftp.ncbi.nlm.nih.gov/blast/db/), Swiss-Prot (A manually annotated and reviewed protein sequence database), GO (Gene Ontology, http://www.geneontology.org/), COG/KOG (Clusters of Orthologous Groups of Proteins/ euKaryotic Ortholog Groups, https://www.ncbi.nlm.nih.gov/research/cog-project/), Trembl (a variety of new documentation files and the creation of TrEMBL, a computer annotated supplement to SWISS-PROT, http://www.ebi.ac.uk/uniprot), and Pfam (Protein Family, https://pfam.sanger.ac.uk/) databases. Fragments per kilobase of transcript per million fragments mapped (FPKM) was used as the gene expression level. The differentially expressed genes (DEGs) were detected by DESeq2 (Love et al., 2014; Varet et al., 2016). Those genes with a |log2Fold Change| ≥ 1 and false discovery rate (FDR) < 0.05 were thought to be DEGs. Gene ontology (GO) enrichment and KEGG pathway analysis of the DEGs were performed.

The metabolites were isolated and detected following the laboratory protocol (Metware Biotechnology Co., Ltd.). The raw data were analyzed with Analyst 1.6.3 software and metware databases to determine and quantify the metabolites. Principal component analysis (PCA), hierarchical cluster analysis, and calculation of Pearson correlation coefficients were performed. The significantly regulated metabolites between groups were determined by variable important in projection (VIP) ≥ 1 and |log2Fold Change| ≥ 1. The variable important in projection values were extracted from the orthogonal partial least squares discriminant analysis (OPLS-DA) result, which was generated using the R 3.5.1 OPLS-DA MetaboAnalystR 1.0.1 (Chong and Xia, 2018). The identified metabolites were annotated using KEGG compound databases and then mapped to the KEGG pathway database. Finally, *K*-means and KEGG enrichment analyses were performed for the significantly differenced metabolites.

### RT-qPCR Analysis

The reliability of RNA-seq was detected with quantitative reverse transcription PCR (RT-qPCR). Complementary DNA (cDNA) was generated with the EasyScript^®^ One-step gDNA Removal and cDNA Synthesis SuperMix kit (Transgen, <city>Beijing </city>, China), and RT-qPCR was performed with FastStart Universal SYBR Green Master (ROX) kit (Roche, Mannheim, Germany) on the ABI QuantStudio 6 Flex instrument according to the instructions of the manufacturer. The tubulin (Supplementary Table 3) gene was used as an internal reference gene. Expression of the 15 genes in the samples (Supplementary Table 3) was detected. Pearson correlation coefficient of log_2_ (fold change of RNA-seq) and log_2_ (fold change of RT-qPCR) was calculated.

### Quantification of ABA and Me-JA by Enzyme-Linked Immune Sorbent Assay

Methods for extraction and purification of ABA and methyl jasmonate (Me-JA) were conducted based on the study of Yang et al. (2001) with some modifications. Briefly, 0.5 g of fresh samples were homogenized and extracted in 4 mL of 80% methanol containing 1 mM butylated hydroxytoluene (BHT) as an antioxidant. The extract was incubated at 4°C for 4 h and then centrifuged at 3,500 rpm for 8 min. The supernatant was transferred to a test tube. Following this method, 1 mL of extraction solution was added to the precipitation and incubated at 4°C for another 1 h, and then centrifuged. The supernatant was combined with that harvested at the first centrifuge, passed through Chromosep C18 columns, and then dried with nitrogen (N) gas. The dried samples were dissolved in 1 mL phosphate buffer saline (PBS) containing 0.1% (v/v) Tween-20 and 0.1% (w/v) gelatin (pH7.5) for analysis by ELISA. The mouse monoclonal antigens and antibodies against ABA and Me-JA and IgG-horseradish peroxidase used in ELISA were produced at China Agricultural University, (<city>Beijing </city>, China). The ELISA analysis method was described in the study of Yang et al. (2001). The calculation of ELISA data was performed following the study of Weiler et al. (1981).

### Analysis of Global DNA Methylation

Genome DNA of different plant materials was extracted by using the cetyltrimethylammonium bromide (CTAB) method according to the study of Doyle and Doyle (1990). The genome DNA was then treated with RNase A (10 mg/mL) (R4875, Sigma-Aldrich, Sint Louis, USA) for 30 min at 37°C, after which the DNA was precipitated with precooled alcohol and dissolved in deionized water. The DNA solution was then mixed with the same volume of 70% perchloric acid and incubated at 100°C for 80 min. The hydrolyzed solution was subsequently neutralized with 5 M KOH. The supernatant of the hydrolyzed solution was obtained after centrifugation at 12,000 rpm for 10 min. The pellets of the first centrifugation were washed with deionized water, and the supernatant was isolated by centrifugation. The two supernatants were mixed and used for high-performance liquid chromatography (HPLC) detection of the content of 5-methylcytosine (5 mC) and cytosine (C). The detection was performed according to the study of Gao et al. (2019) on the instrument Waters E2695 (Milford, America) with a C18 column (Phenomenex 00G-4252-E0, 250 mm × 4.6 mm, 5 μm). The content of 5 mC was calculated according to the equation: 5 mC (%) $=\frac{\;5\text{ mC}}{\left(5\text{ mC}+\text{C}\right)\times \;100\%}$.

### Statistic Analysis

All statistical analysis was obtained from at least three independent replicates (*N* ≥ 3), subjected to two-way analysis of variance. Duncan's multiple range test was compared with the mean differences using the SPSS 20 (IBM Corp., Armonk, NY) software. Differences with *P* < 0.05 were considered significant.

## Results

### Immature SEs of White Spruce Demonstrated Cryotolerance and Embryogenicity After Cold Acclimation

**Figure 1:**
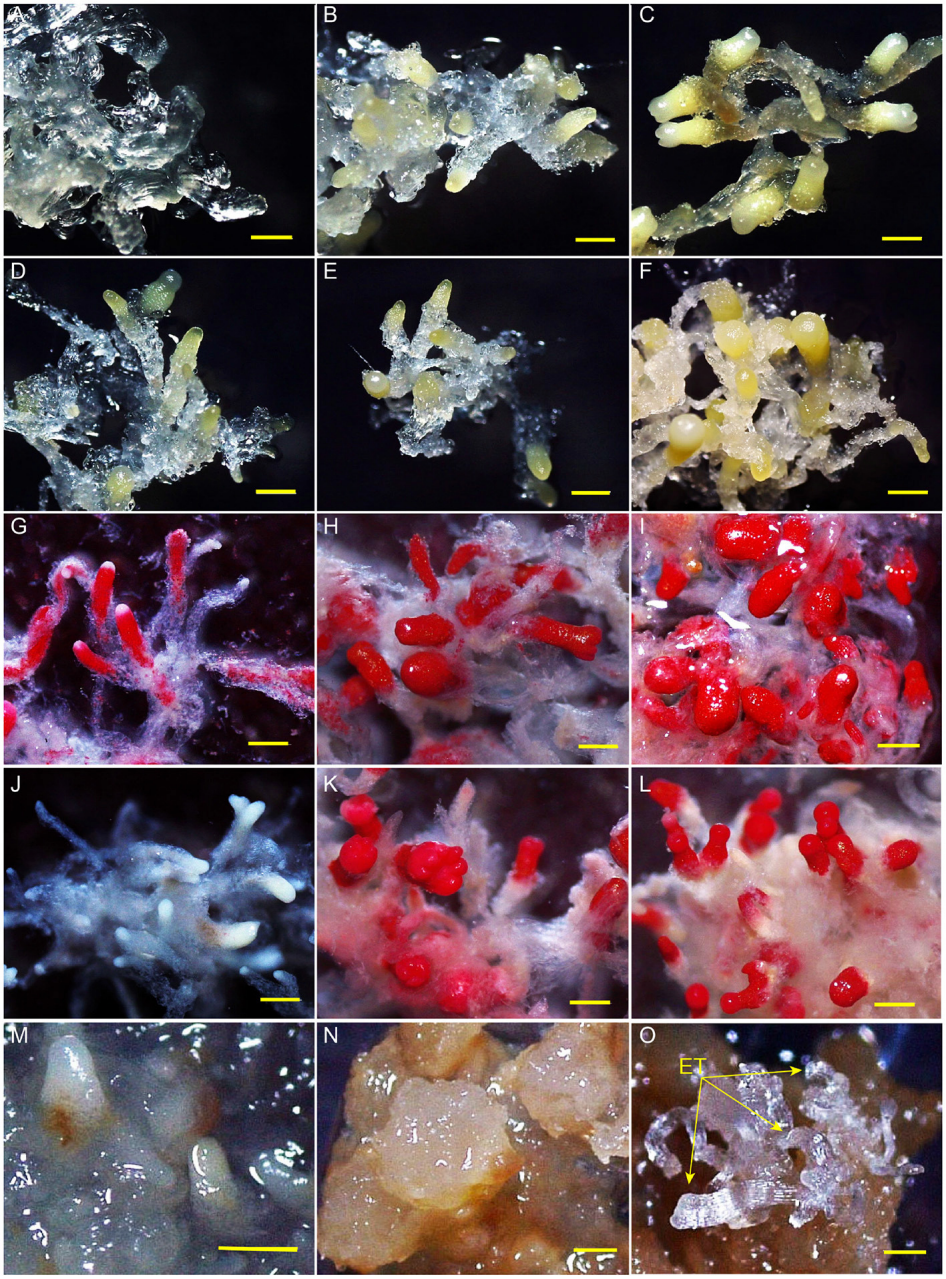
Somatic embryos after various treatments. Development of somatic embryos on maturation medium under 23°C for 0, 2, and 4 weeks were, respectively shown in **(A–C)**. The immature somatic embryos that were cultured on maturation medium at 23°C for 2 weeks and then at 4°C for 2, 4, and 10 weeks were each shown in **(D–F)**. TTC assay of the tissue of MMA2, MMA4 and MCA10 before liquid nitrogen (LN) treatment were, respectively shown in **(G–I)**, and the corresponding results for the tissues after LN treatment were shown in **(J–L)**. Embryogenicity of the tissue of MMA2, MMA4 and MCA10 after LN treatment were, respectively shown in **(M–O)**. The arrow indicates newly re-initiated embryogenic tissue (ET). All bars = 500 μm.

After embryogenic tissues were placed on a maturation medium at 23°C, SEs developed from early immature embryos (0 and 2 weeks) to cotyledonary embryos in 4 weeks (Figures 1A–C). Embryo development was severely stagnated when the cultures were transferred from 23 to 4°C at week 2. No cotyledon formation was observed even after a 10-week-culture at 4°C (Figures 1D–F). All the tissues of MMA2, MMA4, and MCA10 showed cell viability before LN treatment (Figures 1G–I), but only MMA4 and MCA10 demonstrated different degrees of cell viability after LN treatment by TTC assay (Figures 1J–L). The tissue viability after LN treatment was confirmed by transferring the treated tissue onto the induction medium, in which both MMA4 and MCA10 showed a high cryotolerance with a tissue survival rate of nearly 100% (Supplementary Table 2). Although the cotyledonary SEs of MMA4 demonstrated high cryotolerance, their embryogenicity was extremely low (Supplementary Table 2). While tissue acclimated at 4°C for 10 weeks showed the highest induction rate of embryogenic tissue, or embryogenicity, i.e., 91.7% on cryovials or 29.3% on plant tissue (Supplementary Tables 1, 2). Tissue re-growth of MMA2, MMA4, and MCA10 after LN storage is shown in Figures 1M–O. Semi-transparent embryogenic tissue was initiated from the tissue of MAC10 after LN treatment (Figure 1O), and 450 ± 63 SEs produced from one gram of these newly initiated embryogenic tissues, which was comparable to that of the original WSP3 embryogenic tissue. The original WSP3 embryogenic tissue could produce 467 ± 137 SEs per gram of tissue.

### Transcriptome Analysis

**Figure 2:**
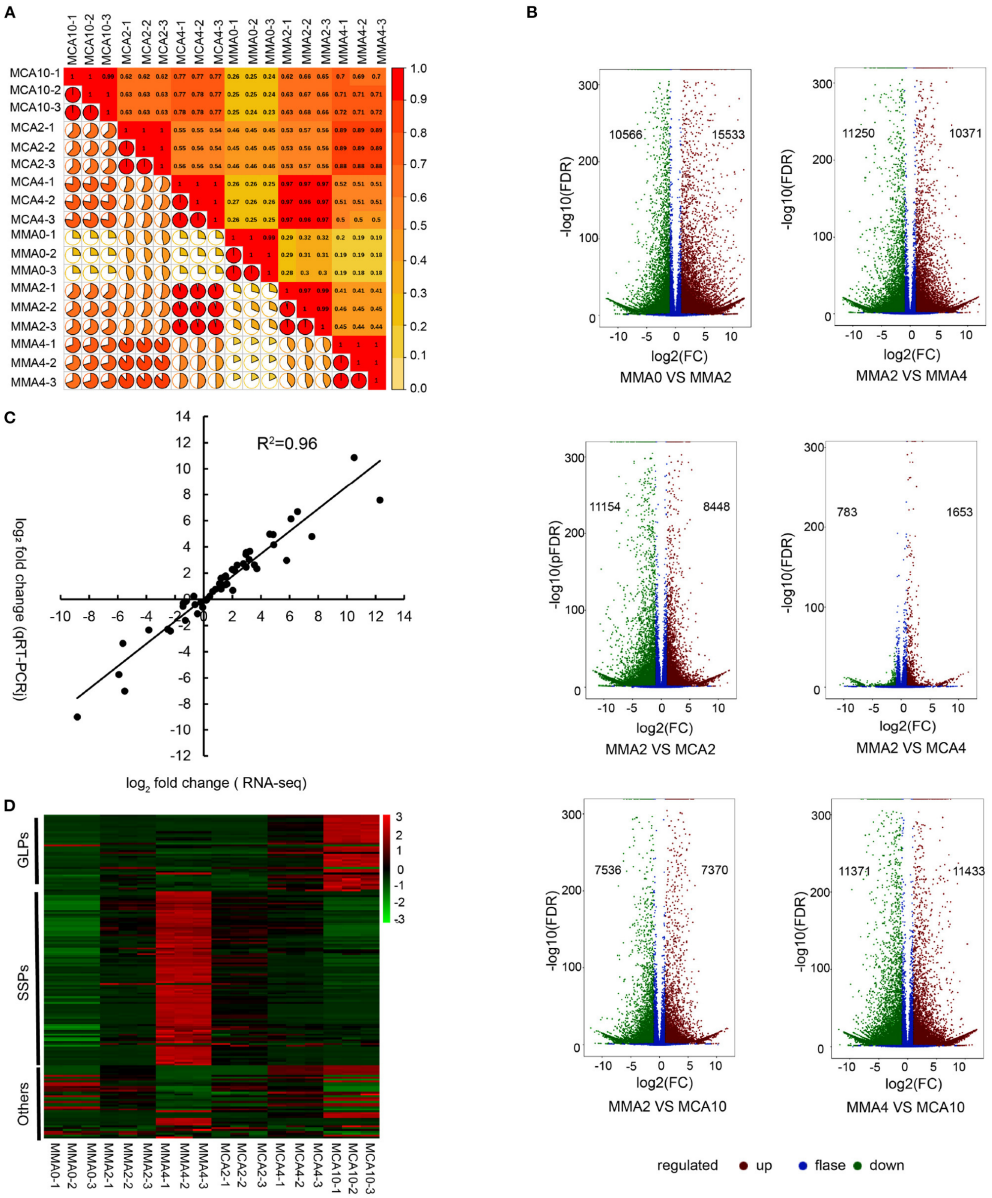
Transcriptome analysis. **(A)** Heatmap of the Pearson's correlation coefficient of different samples. **(B)** Volcano plot of DEGs of MMA0 vs. MMA2, MMA2 vs. MMA4, MMA2 vs. MCA2, MMA2 vs. MCA4, MMA2 vs. MCA10 and MMA4 vs. MCA10. **(C)** Comparison between the log_2_ of gene expression ratios obtained from RNA-seq and qRT-PCR. **(D)** Expression of DEGs enriched in the GO term “nutrient reservoir activity.” GLPs, germin-like proteins; SSPs, seed storage proteins, mainly including albumin-like storage proteins, legumin-like storage proteins, and vicilin-like storage proteins; Others, other proteins, mainly including xylanase inhibitor C-terminal and xylanase inhibitor N-terminal.

A total of 123.99 Gb clean data was obtained. The Clean Data of each sample was more than 6 Gb and the Q30 of them was more than 93%. Pearson's correlation coefficient analysis was conducted to determine gene expression reliability among the samples. The result showed high producibility with the biological replicates (Figure 2A). After the *de novo* transcriptome assembly, a total of 297,374 transcripts were obtained and 230,139 unigenes were further determined. Differentially expressed genes of MMA0 vs. MMA2, MMA2 vs. MMA4, MMA2 vs. MCA2, MMA2 vs. MCA4, MMA2 vs. MCA10, and MMA4 vs. MCA10 were carefully analyzed. The results indicate that both the cold acclimation treatment and the prolonged maturation time could lead to a drastic change in gene expression (Figure 2B). Analysis of qRT-PCR was performed to verify the reliability of RNA-seq. The Pearson correlation coefficient of log_2_ (fold change of RNA-seq) and log_2_ (fold change of RT-qPCR) was 0.96, indicating the reliability of RNA-seq (Figure 2C).

Since both the tissues of MMA4 and MCA10 showed the highest cryotolerance (100%) and of different embryogenicity, DEGs were focused on MMA2 vs. MMA4, MMA2 vs. MCA10, and MMA4 vs. MCA10. The DEGs of MMA2 vs. MMA4, MMA2 vs. MCA10, and MMA4 vs. MCA10 were, respectively 21,621, 14,906, and 22,804, and were classified into 56, 56, and 54 GO terms. The five most significantly enriched GO terms in enrichment hierarchical analysis of DEGs were shown in Table 1. The DEGs of MMA2 vs. MCA10, MMA2 vs. MMA4, and MMA4 vs. MCA10 were significantly enriched in some terms in the biology process. Comparing MMA2 and MCA10, DEGs were most significantly enriched in the metabolism of some antioxidants, such as anthocyanin and phenol-containing compounds, while DEGs for MMA2 and MMA4 were significantly enriched in the metabolism of cell wall constituents, such as amino sugar, chitin, aminoglycan, etc. Differentially expressed genes of MMA4 vs. MCA10 were significantly enriched in the process related to redox reaction, such as oxylipin biosynthetic process, glutathione metabolic process, hydrogen peroxide catabolic process, and hormone catabolic process. In cellular component and molecular function, the DEGs of MMA2 vs. MCA10, MMA2 vs. MMA4, and MMA4 vs. MCA10 were significantly enriched in some common terms, such as lipid droplet, extracellular region part, extracellular space, anchored component of the plasma membrane, and nutrient reservoir activity (Table 1). Although the DEGs of MMA2 vs. MCA10 and MMA2 vs. MMA4 were enriched in some common terms, DEGs might be regulated differently (Figure 2D). Heatmap of these genes showed that expression levels of the album-like storage proteins, legumin-like storage proteins, and vicilin-like storage proteins were gradually up-regulated during SE development at 23°C, down-regulated in the process of 4°C treatment, whereas germin-like proteins (GLPs) were gradually up-regulated during cold acclimation (Figure 2D).

**Table 1:** The five most significantly enriched GO terms in the enrichment hierarchical analysis of DEGs.

**Group**	**Biological process**	**Cellular component**	**Molecular function**
TMA2_vs._TCA10	anthocyanin-containing compound biosynthetic process;phenol-containing compound metabolic process;hemicellulose metabolic process;xyloglucan metabolic process;cell wall macromolecule catabolic process	lipid droplet;anchored component of plasma membrane;extracellular region part;extracellular space;monolayer-surrounded lipid storage body	nutrient reservoir activity;leucocyanidin oxygenase activity;naringenin-chalcone synthase activity;xyloglucan:xyloglucosyl transferase activity;oxidoreductase activity (NAD(P)H as one donor)
TMA2_vs._TMA4	amino sugar catabolic process;chitin metabolic process;chitin catabolic process;aminoglycan catabolic process;cell wall macromolecule catabolic process	lipid droplet;central vacuole;anchored component of plasma membrane;extracellular region part;extracellular space	oxidoreductase activity (NAD(P)H as one donor);oxidoreductase activity, (2-oxoglutarate as one donor);naringenin-chalcone synthase activity;chitinase activity;nutrient reservoir activity
TMA4_vs._TCA10	oxylipin biosynthetic process;organic hydroxy compound catabolic process;hormone catabolic process;glutathione metabolic process;hydrogen peroxide catabolic process	extracellular region part;extracellular space;cell surface;anchored component of plasma membrane;lipid droplet	oxidoreductase activity (NAD(P)H as one donor);glutathione transferase activity;beta-glucosidase activity;manganese ion binding;nutrient reservoir activity

### Metabolome Analysis

**Figure 3:**
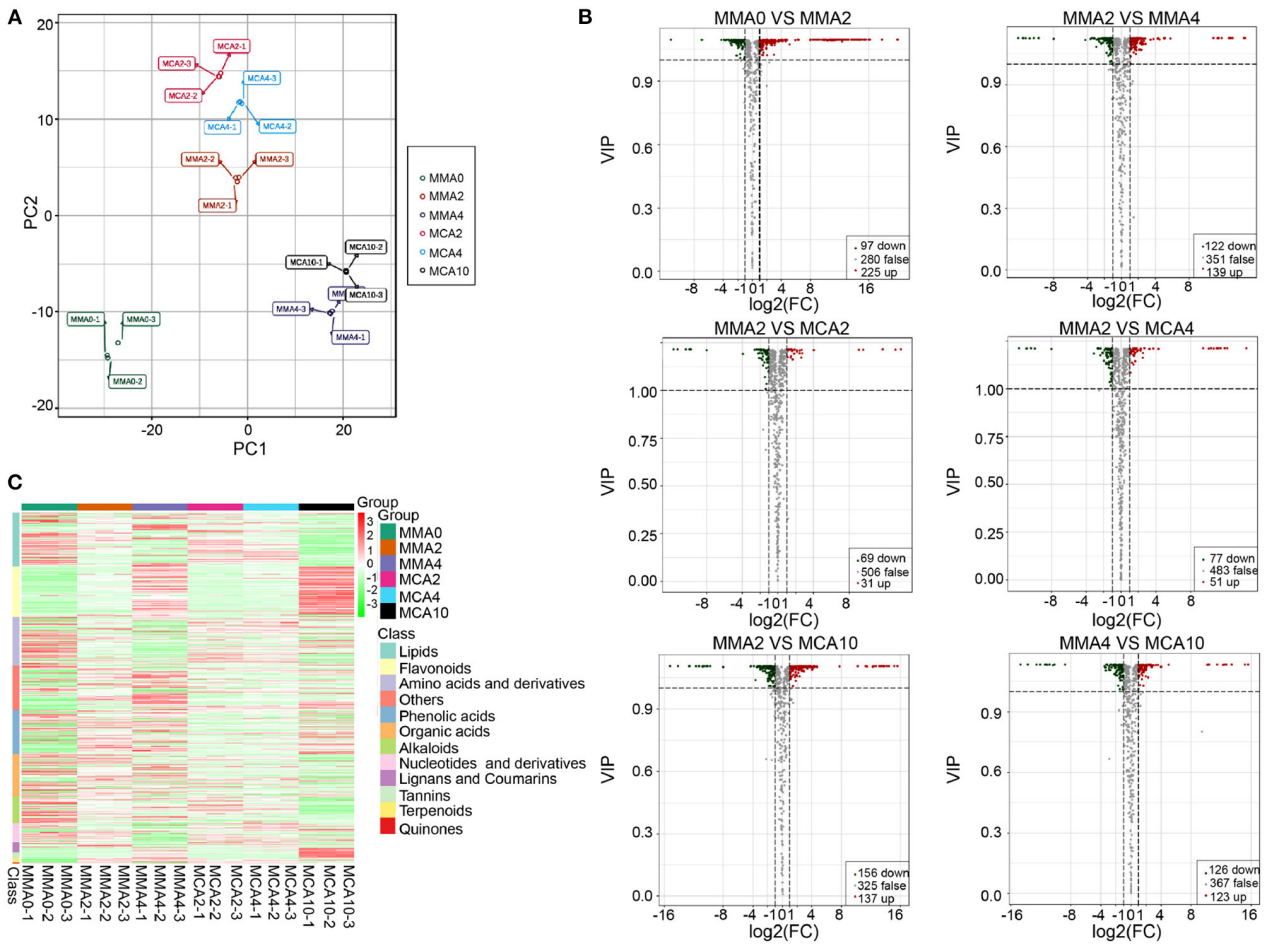
Metabolome analysis. **(A)** Score plot of the principal component analysis. **(B)** Volcano plot of the significantly different metabolites. **(C)** Heatmap of the significantly different metabolites.

A total of 619 metabolites were detected from the six experimental treatments. The principal component analysis showed samples in the same group were aggregated together, while samples in the different groups were separated, indicating significant changes of metabolites in different groups (Figure 3A). Orthogonal partial least squares discriminant analysis revealed that there were 322, 261, 100, 128, 293, and 249 significantly varied metabolites in MMA0 vs. MMA2, MMA2 vs. MMA4, MMA2 vs. MCA2, MMA2 vs. MCA4, MMA2 vs. MCA10, and MMA4 vs. MCA10, respectively. The detailed information about the significantly up- and down-regulated metabolites is shown in Figure 3B.

Hierarchical cluster analysis of the significantly different metabolites (Figure 3C) showed that during the maturation process (23°C), most flavonoids, sugars, and sugar alcohols were gradually up-regulated, whereas most amino acids, nucleotides, and as the derivatives, were down-regulated gradually. In contrast, the other sugars, sugar alcohols, amino acids, and their derivatives, varied in a completely different way in the process of cold acclimation (4°C), although most flavonoids, nucleotides, and their derivatives, changed similarly to those in the process of normal maturation. Regulations of lipid metabolism during maturation and cold acclimation also differed from each other. During the process of maturation, some lipids were up-regulated while most lipids were down-regulated during cold acclimation (Figure 3C).

**Figure 4:**
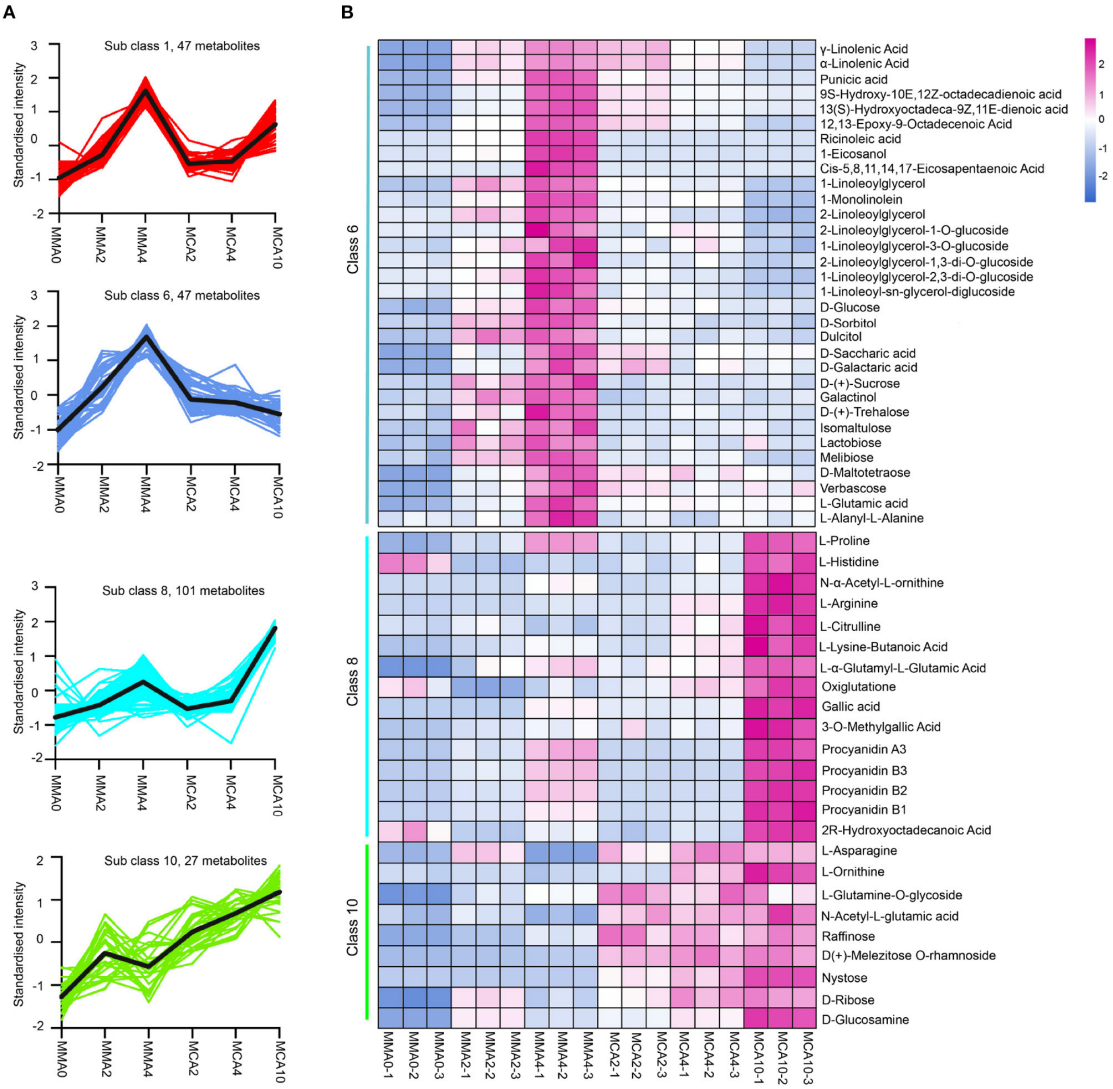
The metabolites up-regulated in MMA4 or MCA10. **(A)** The four sub classes in which metabolites were up-regulated during maturation at 23°C or during cold acclimation at 4°C by *K*-means analysis. **(B)** the heatmap of the metabolites (except flavonoids) in *K*-means sub-class 6, 8, and 10.

The significantly different metabolites by *K*-means analysis could be classified into 12 categories (Supplementary Figure 1). Among them, the metabolites of sub-classes 8 and 10 accumulated remarkably in samples of MCA10, while the metabolites of sub-class 1 and 6 accumulated significantly higher in MMA4 (Figure 4A). Metabolite analysis was then focused on these 4 sub-classes. The metabolites enriched in sub-class 1 were mainly phenolic acids, lignans, and coumarins, whereas the metabolites enriched in sub-classes 6, 8, and 10 included flavonoids, lipids, sugar, and sugar alcohols, amino acids, and their derivatives (Figure 4B). The metabolites in sub-class 8 included L-proline, L-histidine, N-α-acetyl-L-ornithine, L-arginine, L-citrulline, gallic acid, 3-O-methyl gallate, proanthocyanidins A3, B1, B2, and B3, etc. Some metabolites in Class 8, such as proline and gallic acid were also slightly up-regulated during maturation at 23°C, as the content of proline and gallic acid in MMA4 and MCA10 were, respectively 1.9 and 2.3, and 6,963- and 22,053-fold that in MMA2. The metabolites in sub-class 10 included L-ornithine, L-asparagine, raffinose, nystose, etc. As these metabolites were up-regulated significantly in MCA10, they might attribute to the cryotolerance of MCA10 (4°C). The metabolites in sub-class 6 included α-linolenic acid, γ-linolenic acid, ricinoleic acid, and some other unsaturated fatty acids, D-sorbitol, D-saccharic acid, D-Glucose, D-(+)-sucrose, D-(+)-trehalose and so on. Most metabolites of sub-class 6 were accumulated significantly in MMA4, which may result in the high cryotolerance of MMA4 (23°C).

### Effects of Culture Temperatures on Endogenous ABA and Me-JA

**Figure 5:**
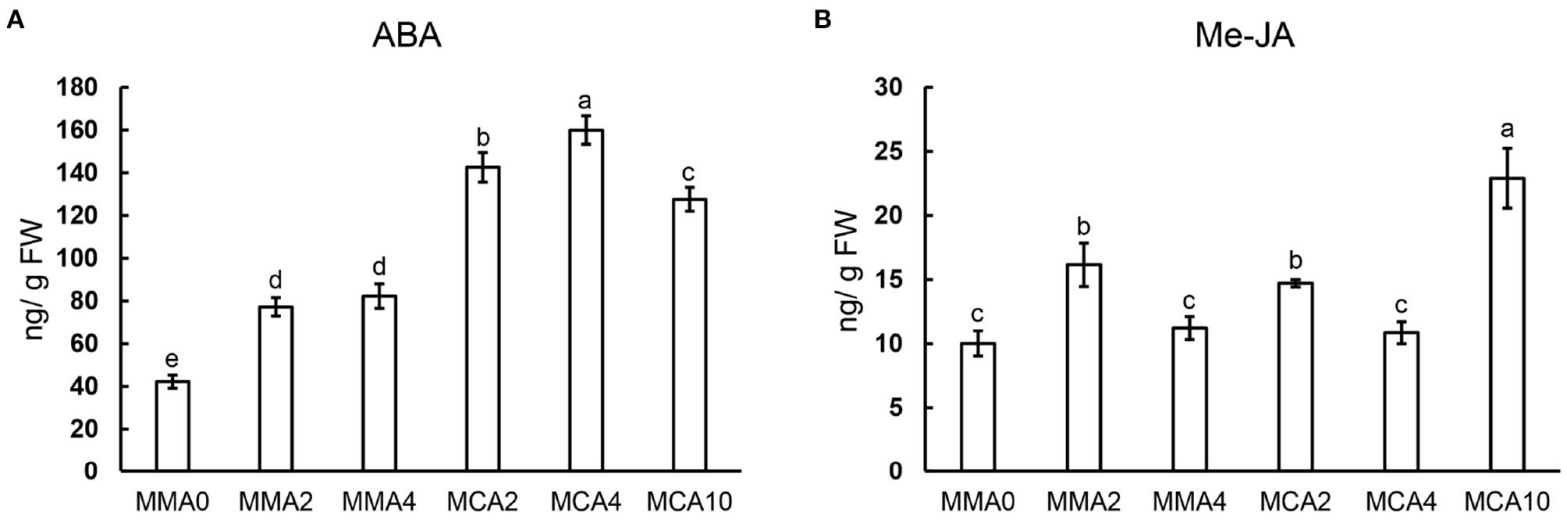
Concentrations of phytohormones in plant tissue following various treatments. **(A)** ABA, **(B)** Me-JA. Significant differences are indicated by different letters (*P* < 0.05). *M* ± *SD, n* = 3.

Concentrations of ABA increased significantly in samples of MCA2, MCA4, and MCA10 during cold treatment (Figure 5A), whereas concentrations of Me-JA fluctuated in samples of different treatments (Figure 5B). The concentration of ABA was higher in MCA10 than those in MMA2 when the cold treatment started, whereas the concentration of Me-JA peaked in samples of MCA10 (Figure 5B). Concentrations of ABA and jasmonic acid increased up to 2- and 1.5-fold, respectively, in the cold-acclimated embryos compared with MMA2.

### The Integrated Transcriptome and Metabolome Analysis

The two-way orthogonal partial least squares (O2PLS) model (Bouhaddani et al., 2016) was established with all the significantly expressed genes and metabolites (Supplementary Figure 2). According to the model, the top 10 genes influencing metabolome included 4 genes coding seed storage proteins, 2 genes coding transcription factors that were related to cell wall synthesis, and 4 other genes which coded RCC2-like protein, linoleate 9S- lipoxygenase, ACC oxidase, and jasmonic acid-amido synthetase genes, respectively. The top 10 metabolites that influenced the transcriptome were vanillin, 12,13-epoxy-9-octadecenoic acid, L-citrulline, L-ornithine, 2-linoleoylglycerol, pinoresinol-4,4'-di-o-glucoside, 1-monolinolein, 1-linoleoylglycerol-2,3-di-o-glucoside, γ-linolenic acid, and punicic acid (9Z,11E,13Z-octadecatrienoic acid), respectively.

**Figure 6:**
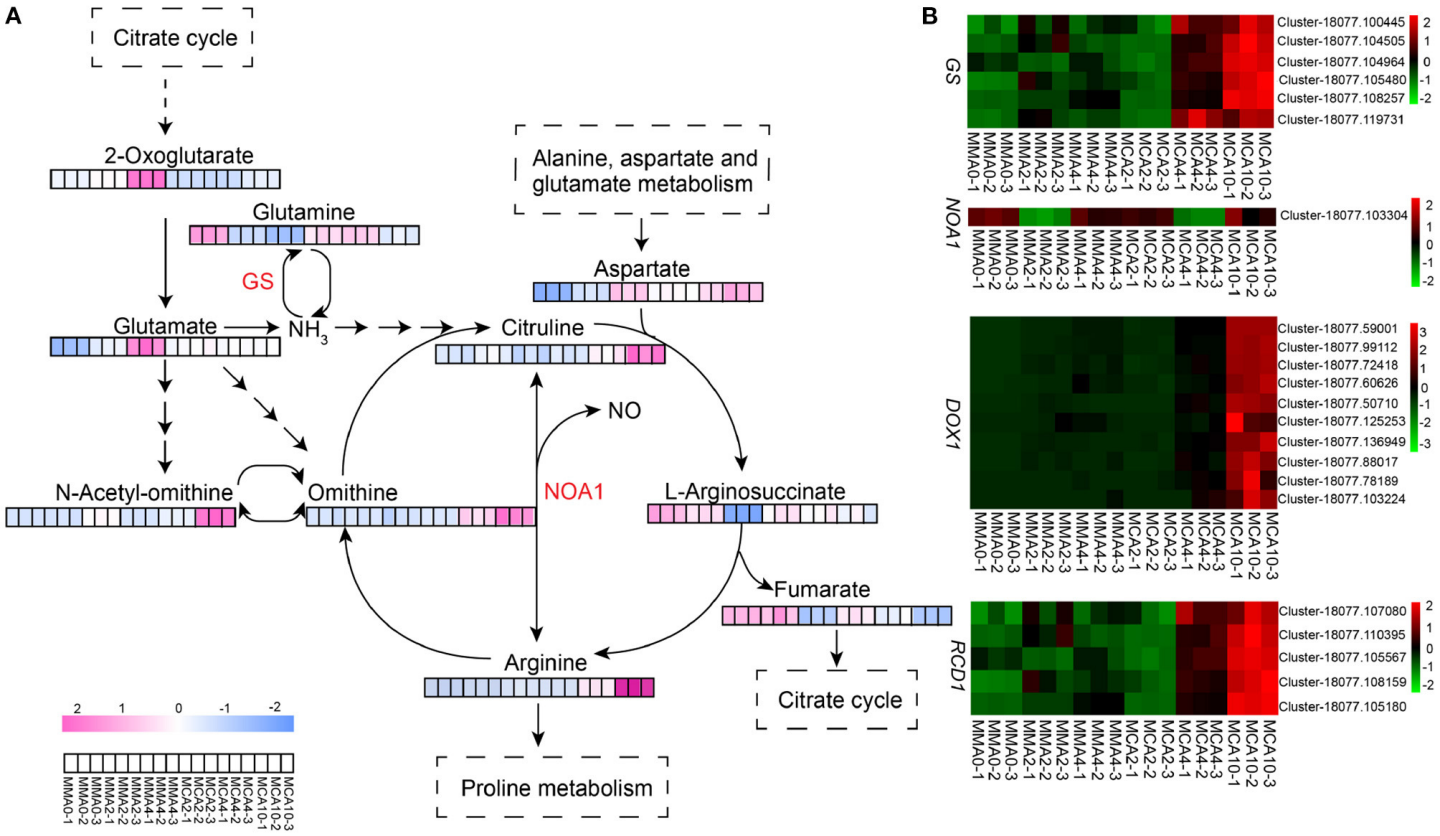
The expression of metabolites and genes related to arginine biosynthesis pathway. **(A)** Heatmap of key metabolites of arginine biosynthesis pathway. **(B)** Heatmap of key genes which might relate to arginine biosynthesis. *GS, glutamine synthetase; NOA1, nitric oxide associated protein 1; DOX1*, α*-dioxygenase gene1; RCD1, radical-induced cell death1*.

The two-way orthogonal partial least squares model showed that L-ornithine and L-citrulline influenced the transcriptome greatly and thus the metabolism study was focused on these two compounds plus L-arginine that could be metabolized into L-ornithine and L-citrulline. These three metabolites accumulated greatly with cold treatment, especially in samples of MCA10 (Figure 6A), which were 8.1, 3.3, and 2.4-fold, respectively of those in MMA2. The expression of *nitric oxide-associated protein 1* (*NOA1*) was up-regulated in MCA10 (Figure 6B) compared with MMA2, which might lead to nitric oxide (NO) production. In the transcriptomic data, the α*-dioxygenase gene* (*DOX1*) was annotated to be a NO response gene. Expression of *DOX1* was highly related to cold treatment (Figure 6B). The α*-dioxygenase gene* was also the main DEGs in the GO term oxylipin biosynthesis, which was one of the most significantly enriched terms on the DEGs of MMA4 vs. MCA10. One of the main catalytic substrates of *DOX1* was linolenic, which was significantly down-regulated during cold treatment (Figure 4B). In contrast, L-arginine, L-ornithine, and L-citrulline were not accumulated significantly in samples of MMA4 when compared with MMA2, while linolenic was significantly enriched in samples of MMA4 (Figure 4B). Two other interesting genes were *glutamine synthetase* (*GS*) and *radical-induced cell death1* (*RCD1*). The integrated analysis of transcriptome and metabolome suggested that *GS* were highly related to L-arginine, L-ornithine, and L-citrulline, and positively regulated these three metabolites, and the expression of *GS* was up-regulated during cold acclimation (Figure 6B). The *radical-induced cell death1* was annotated relating to NO biosynthesis in the transcriptome data and was gradually up-regulated during cold acclimation (Figure 6B).

### Effects of Culture Conditions on Global DNA Methylation of the Embryos

**Figure 7:**
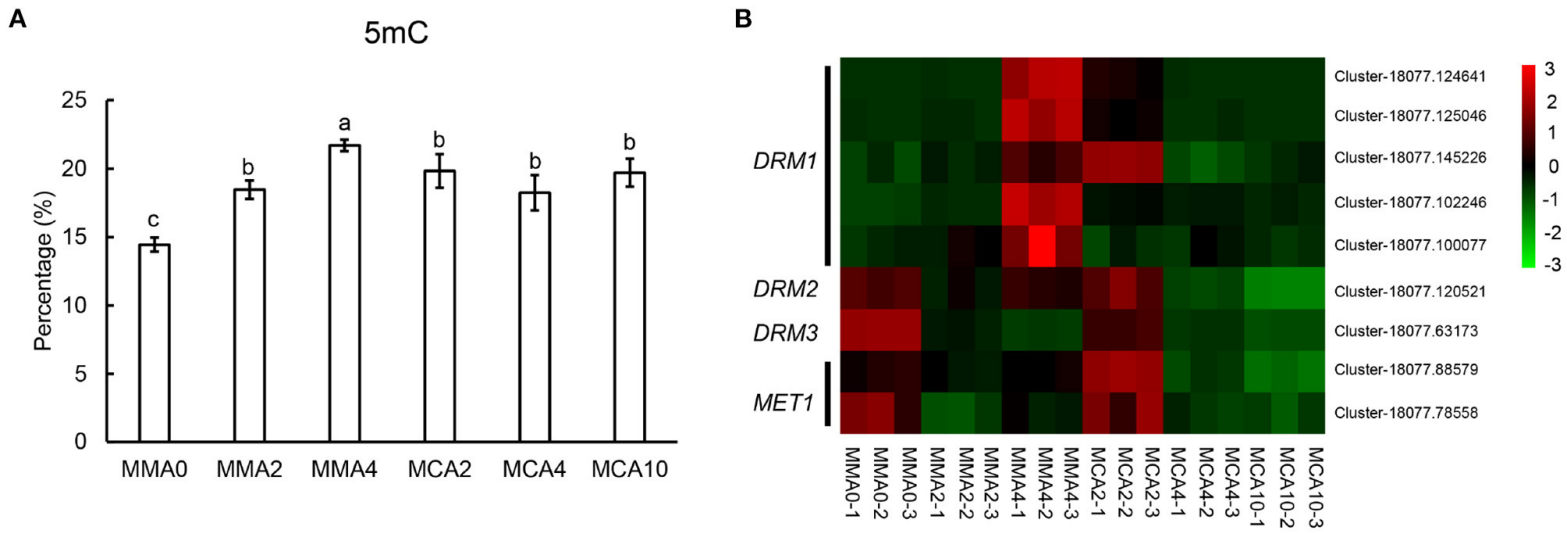
Factors related to embryogenicity. **(A)** Contents of 5 mC showing global methylation levels of tissue of different treatments. **(B)** Heatmap of *DOMAINS REARRANGED METHYLASE (DRM) and DNA methyltransferase 1 (MET1)*. Significant differences are indicated by different letters (*P* < 0.05). Mean ± SD, *n* = 3.

The levels of global DNA methylation increased gradually in plant tissue when SE maturation advanced at 23°C from MMA 2 to MMA4 (Figure 7A). However, little change could be observed in the level of global DNA methylation during cold acclimation treatment at 4°C from MMA 2 to MCA4 or MCA10 (Figure 7A). This was further confirmed with gene expression of *DOMAINS REARRANGED METHYLASE* (*DRM*) and *DNA methyltransferase 1* (*MET1*) (Figure 7B), since RNA-seq data showed that the expression of *DRM* and *MET1*in MCA10 was comparable to that in MMA2.

## Discussion

### Responses of Development and Cold-Tolerance of the Embryos to 23 and 4°C Treatments in *Picea glauca*

Embryogenic tissue of white spruce, as other coniferous species, consists of numerous tiny immature SEs. This tissue (MMA0) could not tolerate cold acclimation treatment at 4°C due to its low cold tolerance. Cold tolerance to 4°C treatment was developed 2 weeks after the tissue was placed on maturation medium and cultured at 23°C (MMA2). Thereafter, with two more weeks cultured at 23°C (MMA4), the embryos could tolerate ultra-low temperature (−196°C), showing a high degree of cryotolerance. This agreed with the previous report (Percy et al., 2001), in which the matured SEs could survive after being preserved in the LN. Cryotolerance of the immature SEs also developed through 10 weeks of cold acclimation after MMA2 treatment, which is consistent with the results of the study conducted by Kong and von Aderkas (2011). Although, both MMA4 treatment and MCA10 treatment improve the cryotolerance of the materials, the development and the embryogenicity of the SEs with the two different treatments were significantly different. Most embryos matured at 23°C for 4 weeks developed cotyledons, while no cotyledon was formed with the embryos under cold acclimation even for 10 weeks. Embryogenicity of the cotyledonary embryos almost vanished but remained by the pre-cotyledonary embryos which underwent cold acclimation. These differences indicate strongly that different mechanisms may exist for building up cryotolerance and retaining embryogenecity in the tissues that underwent 23 or 4°C treatments.

### Metabolite Analysis Reveals Similar and Different Mechanisms Related to Cryotolerance Under Different Temperature Treatments

In samples of MMA4 and MCA10, contents of various oligosaccharides, such as raffinose, D(+)-Melezitose, and O-rhamnoside, were significantly higher than those of MMA2. It is well-known that carbohydrate metabolism is closely related to the antifreeze ability in plants (Atici and Nalbantoglu, 2003). Carbohydrates are also related to osmotic regulation in addition to providing energy. Some sugars and sugar alcohols, have an antioxidant capacity such as galactinol and raffinose (Nishizawa-Yokoi et al., 2008; Sun et al., 2018; Han et al., 2020). Thus, the increased oligosaccharides might contribute to the cryotolerance of both MMA4 and MCA10. Our study also showed that both gallic acid and flavonoids increased significantly during embryo maturation and cold acclimation. Phenolic substances have antioxidant properties protecting plant cells under various stresses. Since both gallic acid and flavonoids are important phenolic substances which play an important role in freezing tolerance (Schulz et al., 2016; Yildiztugay et al., 2017; Sudheeran et al., 2018), these metabolites could enhance cryotolerance. It was notable that proline increased both in the process of maturation and cold acclimation. Proline is closely related to stress resistance in plants. It can be used as a hydroxyl radical scavenger, functioning as an antioxidant (Signorelli et al., 2015). Therefore, this compound might also affect cryotolerance positively to both MMA4 and MCA10.

Sorbitol was often used as an osmolyte in conventional methods of cryopreservation (Chartest et al., 1996). In this study, sorbitol increased gradually in the tissue-cultured at 23°C, whereas it decreased during cold acclimation. Furthermore, KEGG analysis also showed significant differences between samples of MMA4 and MCA10, among the metabolites of sucrose and starch metabolism pathway (Supplementary Figure 3). Samples of MCA10 contained more free monosaccharides and less sucrose than samples of MMA4. In addition, the difference could be observed in metabolites of fatty acids. Unsaturated fatty acids can enhance freezing tolerance in plants since they can adjust the fluidity of cell membranes (Ding et al., 2019). In this study, unsaturated fatty acids increased in MMA4, but decreased in MCA10, when both were compared with MMA2. Less unsaturated fatty acid in MCA10 might result from high expression of *lipoxygenase and DOX1*. This fact implies different mechanisms affecting cryotolerance of either MMA4 or MCA10.

### Transcriptome Analysis Revealed Factors Affecting Cryotolerance

In this study, metabolites in the arginine biosynthesis pathway were indicated to influence the transcriptome significantly. During cold acclimation, metabolites of the arginine synthesis pathway increased significantly. These metabolites are related to the biosynthesis of polyamines, proline, and NO, contributing to freezing tolerance (Shi and Chan, 2013; Winter et al., 2015; Wang et al., 2020). Indeed, the content of proline increased gradually during cold acclimation and peaked in samples of MCA10. Although NO was detected, the transcriptomic analysis showed DEGs of MMA4 and MCA10 were enriched in GO term “cellular response to nitric oxide” (Supplementary Figure 4). Whereas, *DOX1* had been induced by NO triggering plant antioxidant responses (De León et al., 2002). Therefore, the accumulation of L-arginine might result in high expression of *DOX1* in the cold-acclimated samples. Another important gene *RCD1*, which was annotated to be related to NO (Ahlfors et al., 2009; Jaspers et al., 2009, 2010), was up-regulated remarkably. The *radical-induced cell death1* was reported to interact with various transcription factors, such as *MYC2* and *DREB2*, to regulate stress responses (Jaspers et al., 2010), and protect plant cells from redox-imbalances (Hiltscher et al., 2014). High expression of *RCD1* during cold acclimation in this study might help plant cells to avoid oxidative damages caused by the over-production of reactive oxygen species (ROS). When we drew a network with the genes affected by cold acclimation in the STRING database based on *Arabidopsis thaliana* (https://string-db.org) (Szklarczyk et al., 2019), the relationship between the genes related in arginine biosyntheses, such as *GS* and *NOA1*, and the genes responding to NO signal was partially revealed (Supplementary Figure 5).

### Expression of Protein Synthesis Genes, Which Related to Cryotolerance and Embryogenicity Under Different Temperatures

In gene ontology enrichment analysis of DEGs between MMA4 and MCA10, the DEGs in the term of “nutrient reservoir activity” were regulated interestingly. During maturation culture at 23°C, expressions of vicilin-like proteins, legumin-like proteins, albumin storage proteins were gradually up-regulated, indicating a higher maturation degree of the embryos. Differently, during cold acclimation at 4°C, the expression of germ-like proteins (GLPs) was up-regulated. Germ-like proteins are extracellular proteins, they either have superoxide dismutase (SOD) activity or some non-enzymatic biochemical activities. It participates in plant resistance to stresses and regulates plant development (Bernier and Berna, 2001; Lu et al., 2010). Germ-like proteins had been identified in conifer SEs and are considered to be early markers of embryogenesis (Domon et al., 1995; David et al., 1996). In *Pinus caribaea*, the *GLP* gene, *PcGER1*, was specifically expressed in embryos at the early developmental stage, while no *PcGER1* expression was found in non-embryogenic cell lines (Neutelings et al., 1998). In hybrid larch, *LmGER1* showed SOD enzyme activity, regulating cell wall remodeling during SEis (Mathieu et al., 2006).

Most germ-like proteins in this study were annotated to have manganese ion binding activity, suggesting that these proteins may also have SOD enzyme activity. Therefore, we inferred that GLPs in this study might function as antioxidants and play a role in maintaining embryogenicity during cold acclimation. In the previous research, GLP1 (CpGLP1) was regulated in *Craterostigma plantagineum* by ABA, showing SOD activity and controlling cell wall plasticity during desiccation (Giarola et al., 2020). Analyzing the promoter sequences of some *GLPs* genes in this research revealed that there were abundant ABA response elements. As abscisic acid increased significantly during cold acclimation, we speculated that GLPs might be regulated by ABA and contribute to the cell wall plasticity during cryotolerance. The different proteins accumulated in MMA4 and MCA10 not only indicated the degree of embryo maturation but also implied different underlying mechanisms for cryotolerance.

### Endogenous Phytohormones During Cold Acclimation

Plant hormones are thought to be closely related to freezing tolerance in plants. Jasmonic acid (JA) and ABA regulate freezing tolerance through CBF-dependent and CBF-independent pathways, respectively (Shi et al., 2015; Verma et al., 2016). In this study, ABA accumulated significantly during cold acclimation. Abscisic acid concentration in MCA10 was evidently higher than that in MMA2, MMA4, and MCA2. The concentration of JA fluctuated in multiple samples and peaked in samples of MCA10. There might be complex crosstalk between ABA and JA signal pathways to improve the cryotolerance of SEs during cold acclimation, just like what happened under drought stress (De Ollas and Dodd, 2016).

### Factors Affecting Embryogenicity

Results of the transcriptomic and metabonomic analysis revealed accumulations of seed storage proteins, unsaturated fatty acids, and carbohydrates in cotyledonary embryos of MMA4, which indicates a higher degree of embryo maturation than those of MMA2 and MCA10. It is well-known that the degree of embryo maturation corresponds reversely to the embryogenicity of the embryos for initiating embryogenic tissue (Bonga, 2017). This was further supported by the quantities of evidence that genome methylation increased significantly in cotyledonary embryos (MMA4). On the other hand, the cold-acclimated SEs retained their morphology at the earlier developmental stage and high embryogenicity due to less DNA methylation, lower content of storage proteins, and significantly higher expression of embryogenicity-related genes.

## Conclusions

**Figure 8:**
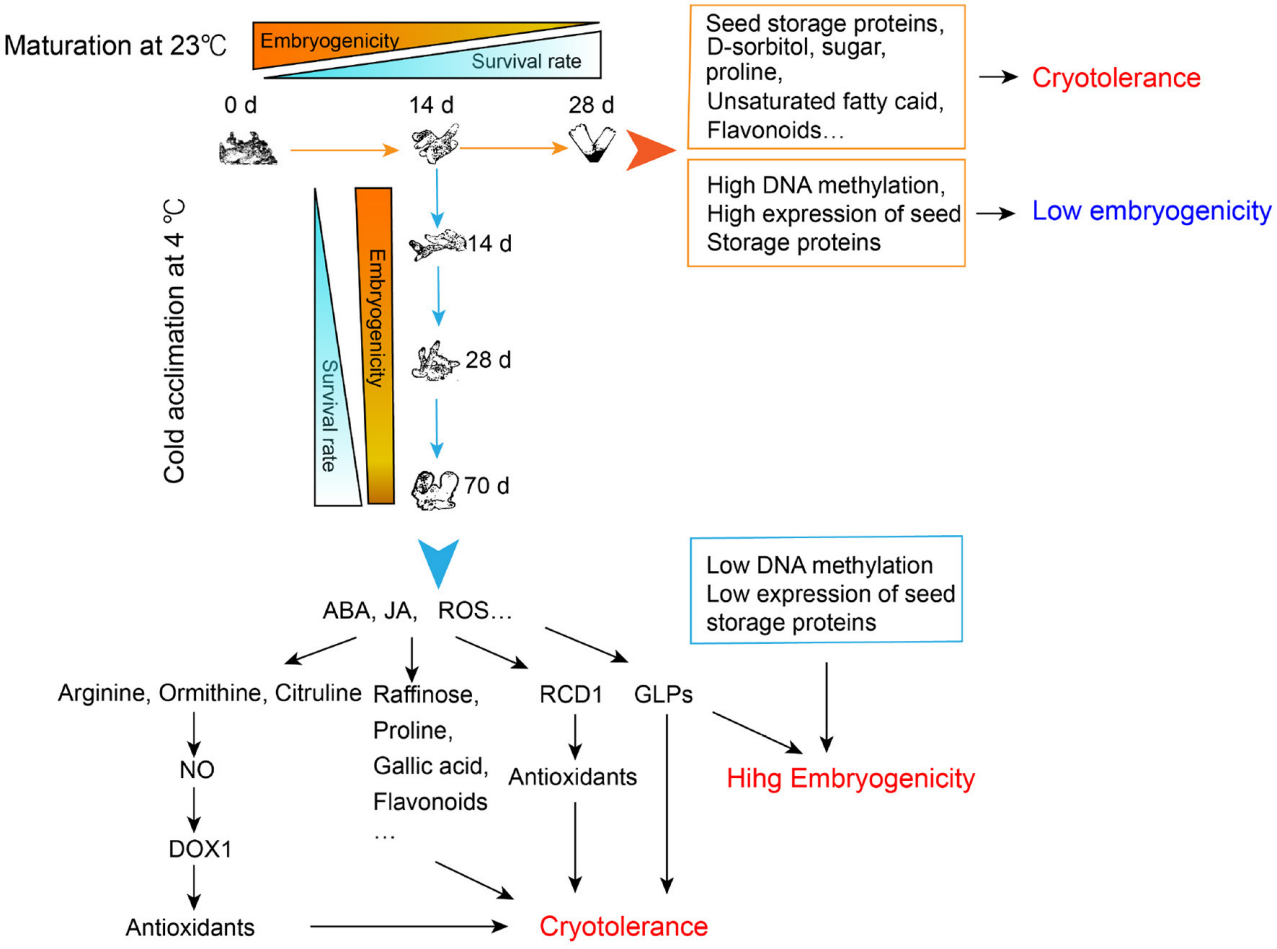
Regulation of cryotolerance and embryogenicity of developing somatic embryos with normal maturation and cold acclimation treatments in *Picea glauca*. ABA, abscisic acid; JA, jasmonate; ROS, reactive oxygen species; NO, nitric oxide; *DOX1*, α-dioxygenase1; RCD1, radical-induced cell death1; GLPs, germin-like proteins.

By performing a cryotolerance test, we confirmed that both the morphologically matured SEs and the cold-acclimated immature SEs could develop their cryotolerance. However, only the latter ones retained high embryogenicity. Based on transcriptomic, metabolomic as well as physiological analysis, we proposed a possible mechanism underlying this phenomenon, which was summarized in Figure 8. Briefly, cryotolerance of SEs matured at 23°C might result from the highly accumulated flavonoids, unsaturated fatty acids, proline, sugars, and seed storage proteins. On the other hand, these embryos almost lost their embryogenicity due to high DNA methylation and advanced embryo maturity, which is reflected by the accumulation of storage proteins. The SEs acclimated at 4°C showed both high cryotolerance and embryogenicity. The high cryotolerance of the cold-acclimated tissue might be mainly due to the significant accumulation of antioxidants, such as raffinose, proline, gallic acid, and flavonoids, whereas the accumulation of which might relate to the high concentrations of ABA, JAin SEs after a 10-week cold acclimation. The significantly accumulated arginine, ornithine, citrulline in the immature SEs during cold acclimation might spark NO signal, and then further activate the expression of *DOX1* to produce antioxidants. In addition, ROS might be overproduced during cold acclimation, which further induced the expression of *RCD1* to reduce the potential oxidative damages to the cells. Accumulation of GLPs with SOD activity might further enhance cryotolerance of the cold-treated SEs by adjusting the redox balance. While the cold-acclimated SEs remained their high embryogenicity due to the little-changed DNA methylation levels and less embryo maturity, which was reflected by the lower amount of storage proteins and higher amount of GLPs, the early marker proteins of embryogenesis.

## Data Availability Statement

The datasets presented in this study can be found in online repositories. The names of the repository/repositories and accession number(s) can be found below: NCBI [accession: PRJNA732083]; Figshare [10.6084/m9.figshare.14650527].

## Author Contributions

LK, YC, JZhang, and JZhao designed the experiments and revised the manuscript. YC, YG, RZ, SQ, and YL performed the experiments. YC analyzed the data and prepared the manuscript draft. All authors read and approved the final manuscript.

## Conflict of Interest

The authors declare that the research was conducted in the absence of any commercial or financial relationships that could be construed as a potential conflict of interest.

## Publisher's Note

All claims expressed in this article are solely those of the authors and do not necessarily represent those of their affiliated organizations, or those of the publisher, the editors and the reviewers. Any product that may be evaluated in this article, or claim that may be made by its manufacturer, is not guaranteed or endorsed by the publisher.
