# Supplementary material for: Transcriptomic, Metabolomic, and Physiological Analyses Reveal That the Culture Temperatures Modulate the Cryotolerance and Embryogenicity of Developing Somatic Embryos in Picea glauca
Source: Front Plant Sci. 2021 Sep 1;12:694229. doi: 10.3389/fpls.2021.694229 (PMC8440983; doi:10.3389/fpls.2021.694229)
Supplement: Supplementary file 1 [file Presentation_1.pdf]

Supplementary materials

1. Supplementary Figures

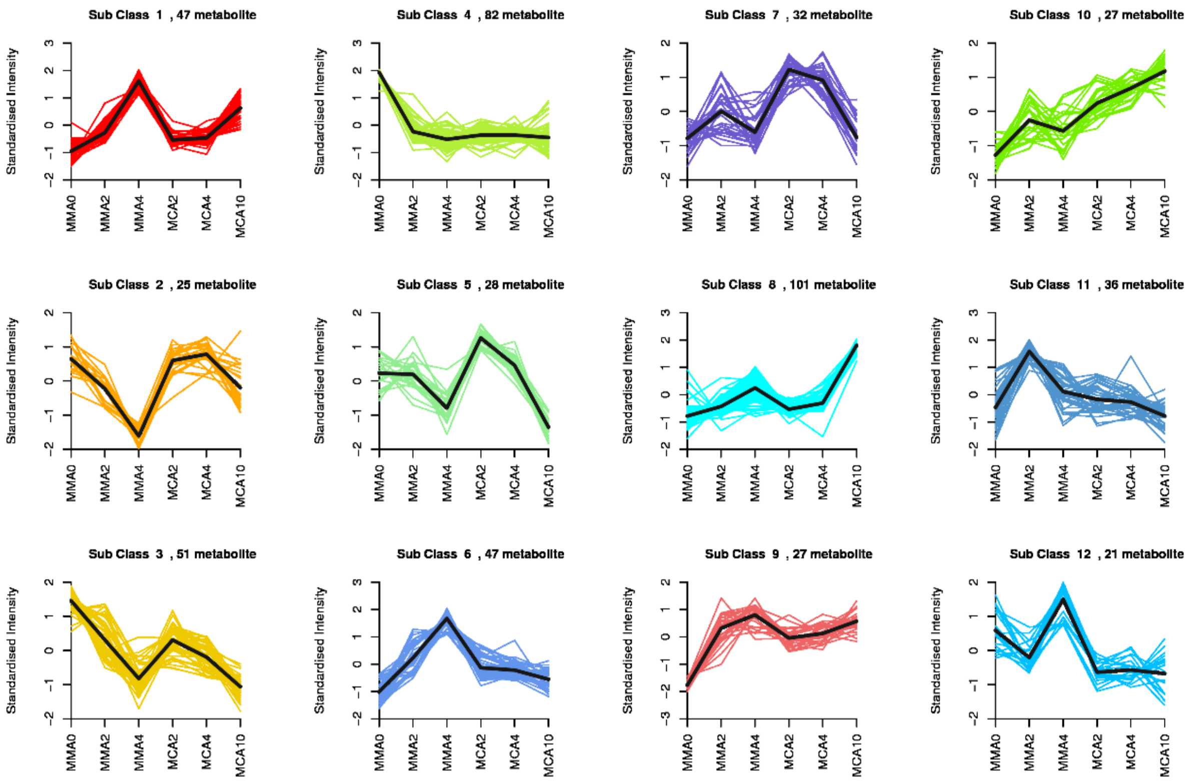

Figure S1. *K*-means analysis of significantly different metabolites

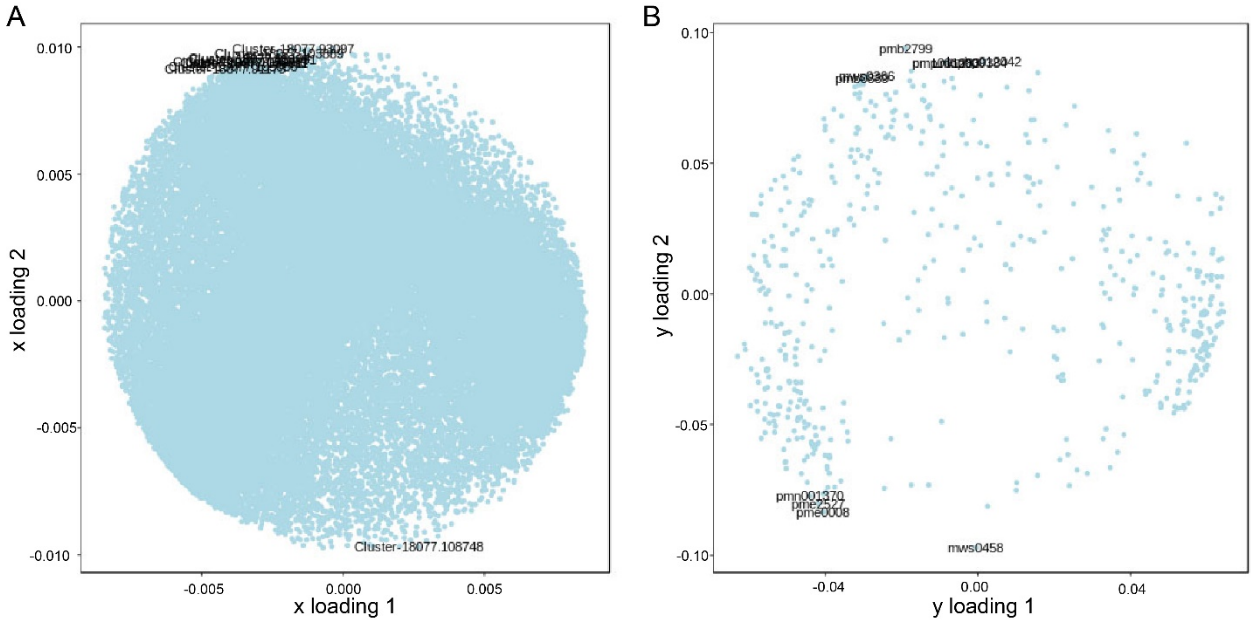

Figure S2. O2PLS model analysis of transcriptome and metabolome a: transcriptome loading plot; b: metabolome loading plot. The distance from each point to the origin point meant the correlation of these transcripts or metabolites with the other omics. The top 10 genes and metabolites that influenced the other omics were indicated in figure S4 a & b respectively.

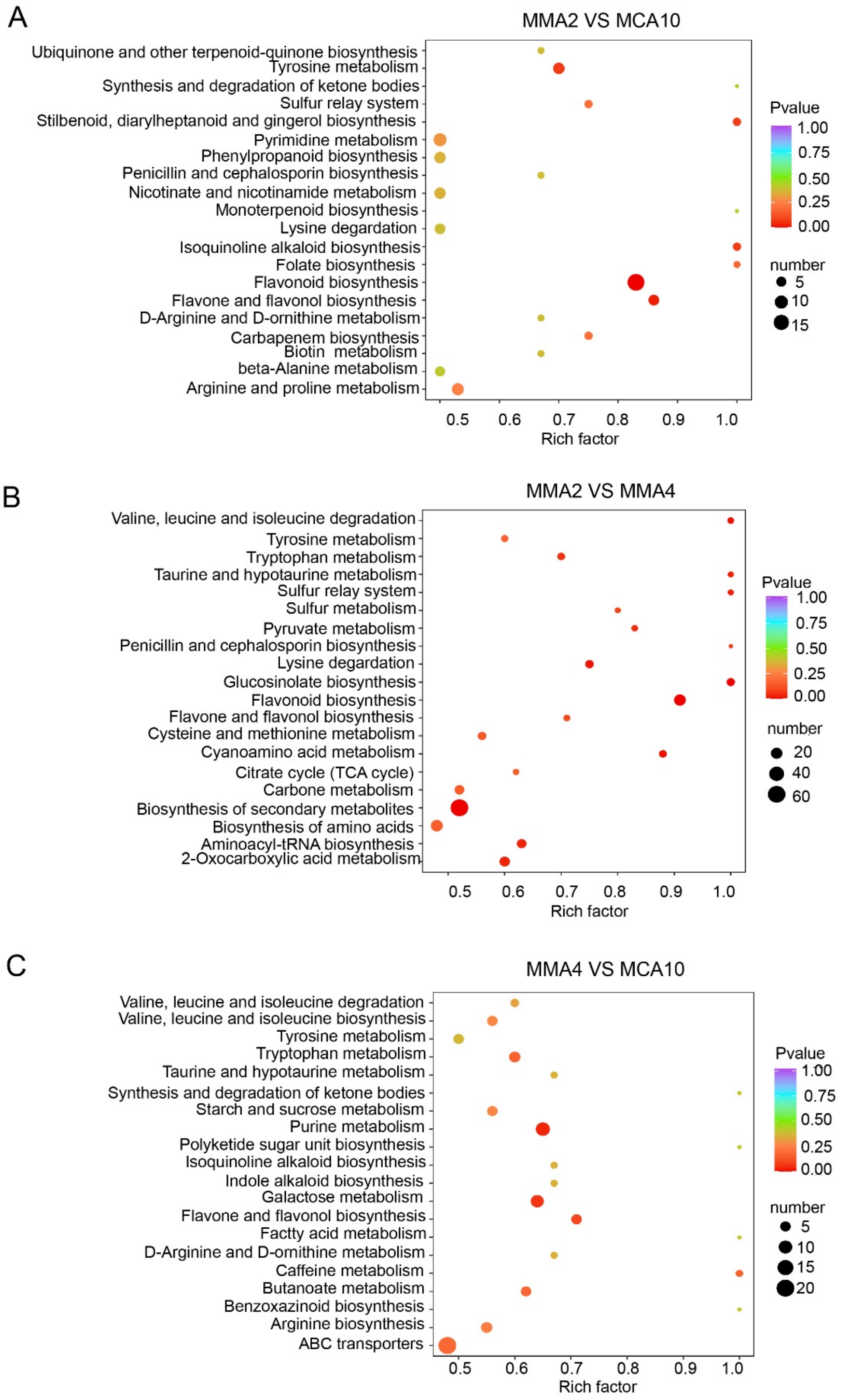

Figure S3. KEGG enrichment pathway analysis. (A) KEGG enrichment pathway of MMA2 vs. MCA10; (B) MMA2 vs. MMA4; (C) MMA4 vs. MCA10.

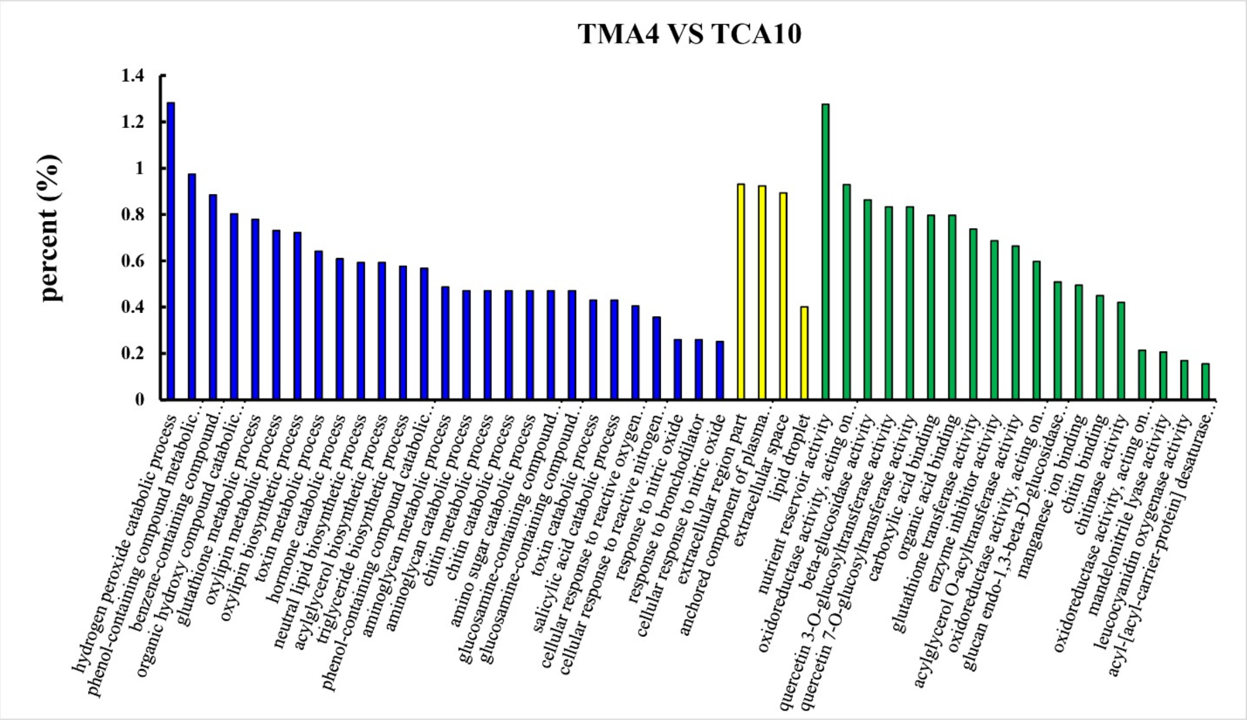

Figure S4. The fifty most enriched GO terms when analyzing DEGs between MMA4 and MCA10

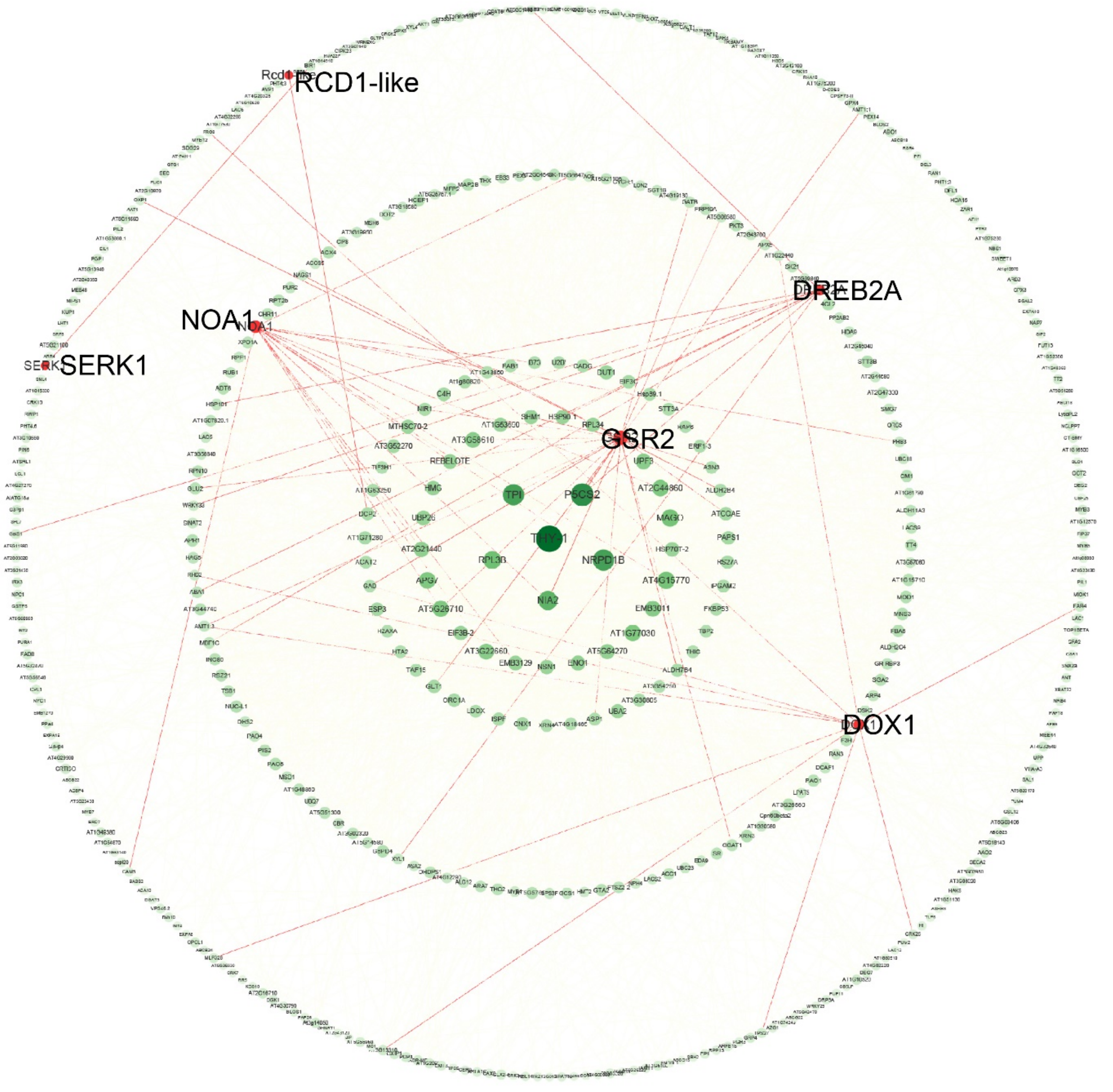

Figure S5. The cytoscape of STRING interactions of the cold acclimation induced proteins. The genes gradually up-regulated during cold acclimation were analyzed with STRING database. A total of 598 proteins were mapped into the database of *Arabidopsis thaliana*, and their STRING interactions with a medium confidence were further revealed by the Cytoscape. *GSR2*, *Glutamine synthetase cytosolic isozyme 1*; *NOA1*, *nitric oxide associated protein 1*; *DOX1*, *α-dioxygenase gene1*; *RCD1-like*, *radical-induced cell death1-like*, *DREB2A*, *dehydration-responsive element-binding protein 2A*; *SERK1*, *Somatic embryogenesis receptor-like kinase 1*.

2. Supplementary Tables

Table S1 Regeneration of embryogenic tissue (ET) from cryo-preserved tissue following various pre-treatments on the basis of cryo-vials. Mean ± SD, *n* = 4.

**Treatment**		**R1**	**R2**	**R 3**	**R 4**	**Total**	**Percentage (%)**
**MMA0**	Vials in test	3	3	3	3	12	0.00±0.00
	Vials of ET regeneration	0	0	0	0	0	
**MMA2**	Vials in test	3	3	3	3	12	0.00±0.00
	Vials of ET regeneration	0	0	0	0	0	
**MMA4**	Vials in test	3	3	3	3	12	8.33±16.65
	Vials of ET regeneration	0	0	0	1	1	
**MCA2**	Vials in test	3	3	3	3	12	8.33±16.65
	Vials of ET regeneration	0	0	0	1	1	
**MCA4**	Vials in test	3	3	3	3	12	24.98±16.65
	Vials of ET regeneration	0	1	1	1	3	
**MCA10**	Vials in test	3	3	3	3	12	91.68±16.65
	Vials of ET regeneration	3	3	2	3	11	

Table S2 Tissue survival rate and embryogenicity before and/or after cryo-preservation in *Picea glauca*. Significant differences are indicated by different letters ($P < 0.05$). Mean ± SD, $n > 5$.

**Treatment**	**Survival rate after cryo-treatment (%)**	**Embryogenicity (%)**	
		**Before cryo-treatment**	**After cryo-treatment**
MMA0	0.00±0.00 (c)	100.0±0.00 (a)	0.00±0.00 (b)
MMA2	0.00±0.00 (c)	100.0±0.00 (a)	0.00±0.00 (b)
MMA4	100.00±0.00 (a)	0.00±0.00 (b)	4.15±6.49 (b)
MCA2	36.73±14.93(b)	100.0±0.00 (a)	2.00±4.90 (b)
MCA4	41.37±12.37 (b)	100.0±0.00 (a)	4.77±7.41 (b)
MCA10	100.00±0.00 (a)	100.0±0.00 (a)	29.29±13.17 (a)

Table S3 Primers used for RT-qPCR

Primers	sequences
tubulin-F	TGAACTTAATACGAACAGGAA
tubulin-R	TTAGTCAGGTTGGCATTC
NOS-F	GCCTTGGATACTACATTAGA
NOS-R	GACTTATGATGGTGTGGAT
ABI5-F	TTCAAGCCACAGAATCAA
ABI5-R	CCATTAGAGGACAGCATT
ABI3-1F	GAAGGATTGAGGAAGAGAC
ABI3-1R	TTACAGCCAGAAGAGACT
DOX-1F	CGTGATAATGTTGCTTGA
DOX-1R	TGAGTGGAGATATTCGTAAT
RCD1-1F	GCAACTTATCTCCAACAATC
RCD1-1R	CCTACGGCTTATATGAATACA
RCD1-2F	TCGCAGAAGAAGTGAATG
RCD1-2R	TGAATATGGAAGTGAAGAACA
PLT-F	GGTGTATTACTTGCCATCAG
PLT-R	CGGTTCTACTTCAGGACTT
NCA-F	GGACTATTGGATGGTGAAT
NCA-R	TGTTGTTGTGGAAGGATT
GLP-1F	CAGATCAGCGACAAGGTAG
GLP-1R	GCGGAGTAGTGGACATTC
GLP-3F	GTTGTGGAGATCAATGTGTT
GLP-3R	GGAAATCTTGGTGGTCATC
ALBUMIN-F	TAAGTGAGCGACGAAGAA
ALMUMIN-R	GGTGAGCATTGAATGAGAG
Vicilline-F	GGAGCATCACCGTATTGG
Vicilline-R	GGAGGTCTCTGAACTTCTTG
thaumin-F	CAAGGTGATCTTCAAGGG
thaumin-R	GGAGTTCACACTCAATGG
DMR2-F	TGATTGAGAAGGAAGATGTT
DMR2-R	CTGCCACCAATAATGAGA
PAL-1F	AAGTCCTCGTAGATGAAT
PAL-1R	GATAATACTCCAGAAGCAT
